# Spontaneous Cerebrospinal Fluid Otorrhea Secondary to Tegmen Tympani Dehiscence: A Case Report and Literature Review

**DOI:** 10.7759/cureus.91908

**Published:** 2025-09-09

**Authors:** Ahmed Hafez Mousa, Badr E Hafiz, Mohammed Aref

**Affiliations:** 1 Neuroscience, Dubai Health, Dubai, ARE; 2 Neurosurgery, Graduate Medical Education (GME), Mohammed Bin Rashid University, Dubai Health, Dubai, ARE; 3 Neurological Surgery, King Faisal Specialist Hospital and Research Centre, Jeddah, SAU; 4 College of Medicine, Alfaisal University, Riyadh, SAU

**Keywords:** cerebrospinal fluid otorrhea, encephalocele, middle cranial fossa approach, semicircular canal dehiscence, skull base defect, tegmen tympani dehiscence

## Abstract

Spontaneous cerebrospinal fluid (CSF) otorrhea is an uncommon but increasingly recognized condition, particularly in older adults. It most often results from temporal bone defects, such as tegmen tympani dehiscence, which establish abnormal communication between the subarachnoid space and the middle ear or mastoid air cells. Clinical manifestations of CSF otorrhea may range from subtle conductive hearing loss and middle ear effusion to life-threatening recurrent meningitis. High-resolution computed tomography (HRCT) is essential for diagnosis and surgical planning. We are presenting a 66-year-old woman with a history of conservatively managed subdural hematoma, who presented with persistent, clear otorrhea from the left ear. Beta-2 transferrin testing confirmed the presence of CSF. HRCT of the temporal bones demonstrated bilateral tegmen tympani thinning with focal dehiscence, most pronounced on the left, along with a small encephalocele extending into the left mastoid air cells and associated mastoid opacification. Bilateral superior semicircular canal dehiscence was also noted. The patient underwent a left-sided middle cranial fossa approach, which confirmed focal tegmen dehiscence with exposed dura. Postoperatively, the otorrhea resolved, and no intracranial complications occurred. Spontaneous CSF otorrhea secondary to tegmen tympani dehiscence should be considered in older patients presenting with unexplained persistent otorrhea or middle ear effusion. HRCT provides crucial diagnostic information, while the middle cranial fossa approach enables direct repair and durable outcomes. Early recognition and surgical intervention are vital to prevent recurrence and serious complications such as meningitis or hearing loss.

## Introduction

Spontaneous cerebrospinal fluid (CSF) otorrhea is a rare but increasingly recognized clinical entity, particularly in older adults [1]. Unlike post-traumatic or iatrogenic CSF leaks, spontaneous leaks occur in patients without a history of head trauma or ear surgery [1]. These leaks are most commonly associated with defects in the temporal bone that establish an abnormal communication between the subarachnoid space and the middle ear or mastoid air cells. Among these, tegmen tympani dehiscence, a bony defect in the roof of the middle ear cavity, is one of the most frequent anatomical causes [2].

The pathophysiology of spontaneous CSF leaks is multifactorial. Idiopathic intracranial hypertension, chronic pulsatile CSF pressure, congenital thinning of the skull base, and age-related bone resorption are proposed mechanisms [3]. Patients may present with a spectrum of symptoms ranging from subtle conductive hearing loss, tinnitus, or recurrent middle ear effusions to more severe complications, including recurrent meningitis or intracranial infections [1,3].

Early recognition is crucial to prevent morbidity. High-resolution computed tomography (HRCT) provides a detailed evaluation of bony defects and may reveal associated encephaloceles, mastoid opacification, or semicircular canal dehiscence [4]. Magnetic resonance imaging (MRI) can complement CT when soft tissue evaluation or identification of herniated brain tissue is needed. Surgical repair is generally recommended, with the middle fossa approach offering direct visualization of the dural defect and allowing durable multilayered closure [4].

## Case presentation

A 66-year-old woman with a background of conservatively managed spontaneous right fronto-parietal subdural hematoma two years ago, presented with a new complaint of persistent, clear watery discharge from the left ear for seven months. There was no history of trauma, previous ear surgery, or active infection. She was seen by multiple physicians and was missed. Otoscopic examination revealed a fluid-filled middle ear on the left side, and beta-2 transferrin testing confirmed the presence of CSF in the otorrhea.

**Figure 1:**
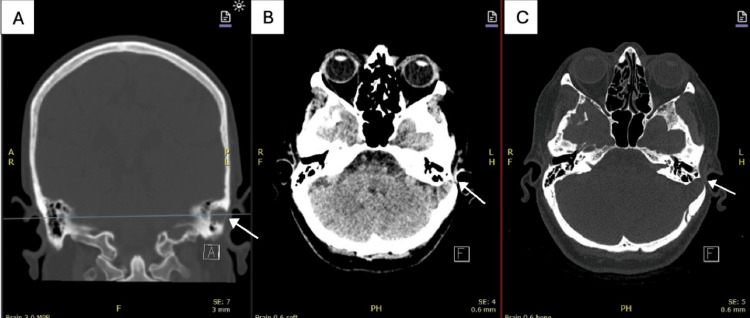
High-resolution computed tomography of the temporal bones. (A) Coronal reconstruction in bone window revealing erosion of the left petrous bone above the labyrinth, confirming tegmen dehiscence with associated cortical thinning with defect and opacification of the mastoid air cells (white arrow). (B) Axial reconstruction in soft tissue window demonstrating a focal bony defect of the left tegmen tympani with soft tissue density herniating into the mastoid air cells (white arrow), consistent with encephalocele and CSF otorrhea. (C) Axial reconstruction in bone window demonstrating a focal bony defect of the left tegmen tympani with soft tissue density herniating into the mastoid air cells (white arrow), consistent with encephalocele and CSF otorrhea.

A high-resolution CT scan of the temporal bones (Figure 1) demonstrated significant thinning and focal dehiscence of the bilateral tegmen tympani, most pronounced on the left side, with a suspected small encephalocele herniating into the left mastoid air cells. There was associated partial opacification of the left mastoid. Additionally, bilateral diffuse dehiscence of the arcuate segments of the superior semicircular canals was observed. On the right side, the middle ear cavity exhibited soft tissue changes but no active CSF leak.

Given the radiological evidence and clinical presentation, the patient underwent a left-sided middle cranial fossa approach for repair of the tegmen defect. Intraoperatively, a focal bony dehiscence with exposed dura was confirmed overlying the tegmen tympani. The encephalocele was carefully reduced, and the defect was closed using a multilayered repair technique with temporalis fascia graft, bone chips, and fibrin sealant. The postoperative period was uneventful, with cessation of the otorrhea and no signs of intracranial complications.

## Discussion

Spontaneous CSF otorrhea due to tegmen tympani dehiscence is a rare but important differential diagnosis in elderly patients presenting with persistent middle ear effusion or clear otorrhea [5]. While the majority of CSF leaks are secondary to trauma or surgical procedures, spontaneous leaks often occur in the context of predisposing anatomical or physiological factors [6]. Idiopathic intracranial hypertension can generate chronic pulsatile pressure on thin temporal bone structures, leading to progressive erosion [6,7]. Congenital bone thinning or age-related osteopenia may further predispose patients to spontaneous defects [7].

CT imaging remains the gold standard for identifying tegmen defects and associated abnormalities such as encephaloceles, mastoid opacification, and semicircular canal dehiscence. MRI may be used adjunctively to evaluate soft tissue herniation [8]. In the presented case, bilateral arcuate dehiscence may represent either a congenital predisposition or a contributing factor to pressure-induced bone erosion, highlighting the importance of a thorough radiological assessment [9].

The middle cranial fossa approach is considered the standard surgical technique for repair of tegmen defects [10]. This approach allows direct visualization of the dural breach, safe reduction of any encephaloceles, and multilayered reconstruction, which may include temporalis fascia, bone grafts, and fibrin sealant. Alternative approaches, such as transmastoid or combined techniques, may be appropriate depending on defect size and location, but MCF repair provides the most durable outcomes. Successful repair reduces the risk of recurrent CSF leakage, meningitis, and conductive hearing loss [10].

Long-term follow-up is essential to monitor for recurrence or complications. Early recognition and timely surgical intervention are critical to improving patient outcomes and preventing potentially life-threatening sequelae [11].

We did an extensive literature review that focuses on reports and series published from 2010 through 2025 that address spontaneous CSF otorrhea and/or temporal bone meningoencephaloceles involving the tegmen. It emphasizes adult spontaneous cases, surgical approach, and outcomes. Cells are marked NR (not reported) when details were not available in accessible abstracts or open texts (Table 1).

**Table 1:** Literature table focusing on reports and series published from 2010 through 2025 that address spontaneous cerebrospinal fluid (CSF) otorrhea and/or temporal bone meningoencephaloceles involving the tegmen. NR: not reported; CT: computed tomography; HRCT: high-resolution computed tomography; MRI: magnetic resonance imaging; MCF: middle cranial fossa; MF: middle fossa; TM: transmastoid; ICP: intracranial pressure; STP: subtotal petrosectomy; AEs: adverse events.

Study (year)	Design/setting	Number (patients)	Defect site focus	Imaging modality	Surgical approach	Graft/materials	Key outcomes (CSF resolution/recurrence)	Follow-up
Markou et al. (2011) [12]	Series: osteodural temporal bone defects	12	Temporal bone (osteodural defects)	CT/MRI	Approach per site (MCF/TM)	NR	Successful closure	NR
Stucken et al. (2012) [13]	Series: obesity & spontaneous temporal bone encephaloceles/CSF leak	NR	Temporal bone encephaloceles (incl. tegmen)	CT/MRI	Varied (MCF/TM)	NR	Association with obesity; effective repair	NR
Kenning et al. (2012) [14]	Series: temporal bone meningoencephaloceles/CSF leak with ICP paradigm	NR	Temporal meningoencephaloceles & CSF leaks	CT/MRI	MCF ± combined; CSF diversion when needed	Varied	Good closure; consider ICP control	NR
Boo et al. (2013) [15]	Case report	1	Tegmen (spontaneous)	CT/MRI; beta-2 transferrin	Transmastoid	Bone chips	Leak resolved; no recurrence	NR
Carlson et al. (2013) [16]	Series: temporal bone encephalocele/CSF fistula repair (MCF/combined)	NR	Temporal bone (MCF or combined)	CT/MRI	MCF or combined TM–MCF	Varied	High success	NR
Kim et al. (2014) [17]	Series: transmastoid outcomes in spontaneous temporal bone CSF leak	NR	Temporal bone spontaneous leaks	CT/MRI	Transmastoid with obliteration	Varied	Good hearing & repair success	NR
Marchioni et al. (2014) [18]	Series: combined TM + minicraniotomy	NR	Tegmen defects (CSF/encephalocele)	CT/MRI	Combined TM + minicraniotomy	Varied	All defects closed; low complications	NR
Ahmed et al. (2017) [10]	Case series	5	Tegmen tympani defects (spontaneous)	HRCT/MRI	Keyhole MCF	Temporalis fascia + bone chips + fibrin	All resolved without recurrence	NR
Thomeer et al. (2020) [19]	Cohort	12	Spontaneous lateral skull base (often tegmen)	CT/MRI; beta-trace/beta-2	MCF (7), TM (3), STP (4); 3 revisions	Varied/obliteration	No recurrence at a mean of 13 months; no severe AEs	Mean 13 months
Ren et al. (2021) [20]	Retrospective series (approach comparison)	NR	Spontaneous CSF otorrhea	CT/MRI	TM vs. MCF vs. combined	NR	TM effective; favorable audiometrics in select cases	NR
Swanson et al. (2022) [21]	Single-center outcomes	NR	Tegmen dehiscence repairs	CT/MRI	MCF with autologous temporalis fascia	Temporalis fascia	High symptomatic improvement; safe	NR
Ellsperman et al. (2024) [22]	Retrospective review (intradural MF ± TM)	NR	Lateral temporal bone encephaloceles/CSF leak	CT/MRI	MCF or MCF+TM intradural reinforcement	Autologous grafts	Long-term success: ~88%; hearing stable/improved: ~94%	NR

## Conclusions

Spontaneous CSF otorrhea is a rare yet clinically significant manifestation of tegmen tympani dehiscence, particularly in older adults without a history of trauma or surgery. This case highlights the importance of maintaining a high index of suspicion in patients presenting with persistent otorrhea and radiological evidence of skull base erosion. High-resolution temporal bone CT is essential for diagnosis and surgical planning. The middle cranial fossa approach remains the definitive method for achieving direct repair of tegmen defects with durable outcomes. Timely recognition and intervention are critical to prevent complications such as recurrent meningitis, hearing loss, or intracranial infections.
